# Patient-specific plate for navigation and fixation of the distal radius: a case series

**DOI:** 10.1007/s11548-021-02320-5

**Published:** 2021-02-11

**Authors:** Johannes G. G. Dobbe, Abbas Peymani, Hendrika A. L. Roos, Maikel Beerens, Geert J. Streekstra, Simon D. Strackee

**Affiliations:** 1 Department of Biomedical Engineering and Physics, Amsterdam Movement Sciences, Amsterdam UMC, University of Amsterdam, Room No L0-113-3, Meibergdreef 9, 1105 AZ Amsterdam, The Netherlands; 2 Department of Plastic, Reconstructive and Hand Surgery, Amsterdam Movement Sciences, Amsterdam UMC, University of Amsterdam, Meibergdreef 9, Amsterdam, The Netherlands; 3 Xilloc Medical, Urmonderbaan 22, Sittard-Geleen, The Netherlands

**Keywords:** Malunion, Corrective osteotomy, 3D planning, Computer-assisted surgery, Custom treatment

## Abstract

**Purpose:**

Corrective osteotomy of a malunited distal radius conventionally relies on 2D imaging techniques for alignment planning and evaluation. However, this approach results in suboptimal bone repositioning, which is associated with poor patient outcomes. In this case series, we evaluate the use of novel patient-specific plates (PSPs), which feature navigation and fixation of bone segments as preoperatively planned in 3D.

**Methods:**

Ten participants with distal radius malunion underwent CT scans for preoperative alignment planning. Patient-specific guides and plates were designed, 3D-printed, and sterilized for use in corrective surgery of the distal radius. Pre- and postoperative results were compared in regard to clinical, functional, and radiographic outcomes.

**Results:**

The application of a PSP was successful in 7 of the 10 cases. After treatment, the residual alignment error was reduced by approximately 50% compared with conventional treatment. The use of PSPs reduced pain significantly. Pre- and postoperative results were pooled and demonstrated significant correlations between: (1) pain and malpositioning, (2) the range of pro- and supination motion, the MHOQ score, the EQ-5D-5L score and dorsovolar angulation, and (3) MHOQ score and proximodistal translation.

**Conclusion:**

The correlation between malalignment and MHOQ score, EQ-5D-5L score, pain, and range of motion shows that alignment should be restored as well as possible. Compared to the conventional approach, which relies on 2D imaging techniques, corrective osteotomy based on 3D preoperative planning and intraoperative fixation with a PSP has been shown to improve bone alignment and reduce pain.

**Level of evidence:**

IV.

## Introduction

Fractures of the distal radius may result in symptomatic malunion of bone segments. Approximately 5% of patients with malunion of the distal radius [1] experience severe symptoms, such as pain and decreased function. To treat the underlying pathology, patients often undergo a corrective osteotomy [2–5]. In this procedure, the surgeon cuts the bone and reduces the two bone segments using a plate, the aim being to improve anatomical alignment. However, positioning may be compromised as conventional T or L plates do not always fit a deformed radius, and often require subjective bending by the surgeon to fit the bone surface. Bending is sometimes objectified by using 3D printed bone models to shape a plate preoperatively [6]. Bone repositioning using an anatomically shaped plate depends on the shape of the plate chosen by the plate manufacturer [7]. Currently, preoperative planning techniques are mainly based on 2D X-ray imaging, and intraoperative positioning is assessed by 2D fluoroscopic imaging. This conventional approach has been shown to result in suboptimal bone repositioning, which in turn is associated with poorer patient outcomes [8].

Over the last few decades, a number of techniques have emerged to plan the repositioning of bone segments preoperatively in 3D [9–21]. The availability of physically printed or virtually displayed bone models allows for a better appreciation of the deformity and helps planning alignment in the coronal, sagittal, and transverse planes. A concomitant challenge of 3D planning is the transfer of the plan to the patient, which also requires an approach in 3D, i.e., restoring three bone translations along, and three bone rotations about the axes of a 3D coordinate system.

A novel approach to transfer the preoperative plan of a corrective distal radius osteotomy to the patient is based on a patient-specific plate (PSP). In this approach, a custom surgical guide is first used to predrill holes and to perform the osteotomy. Subsequently, the predrilled holes are used to fix the bone segments to a PSP using screws. The PSP is designed to fit the patient’s bone contour optimally; this has the advantage of aligning the bone segments according to the preoperative plan, which may limit tendon ruptures [22], and obviating the need for secondary surgery for plate removal [23] as is common when using standard plates. Preclinical studies [15, 20] and initial clinical evaluations [16, 24, 25] have shown the PSP method to be promising for corrective osteotomy of the distal radius.

In this case series, we evaluate the clinical, functional, and radiological outcomes of patient-specific plating in distal radius malunion. We hypothesize that the use of patient-specific plates improves the anatomical alignment of bone segments and therefore clinical outcomes.

## Methods

### Patient selection and scanning

Patients who presented at our outpatient clinic with a malunion of the distal radius between June 2017 and June 2019 were CT scanned, and the deformity was evaluated using 3D modeling technology [13, 15, 16]. Cases judged by the surgeon to be complex were eligible for inclusion in the study if the patient was over 16 years of age and had symptoms of pain, limited range of motion, or decreased hand function. Exclusion criteria were pregnancy, underlying congenital disorders of the upper limb (e.g., Madelung deformity), or a history of fracture of the contralateral bone, since this serves as the reference bone in position planning. Ten patients agreed to participate. This study was performed in line with the principles of the Declaration of Helsinki. Written informed consent was obtained from the patients for their data to be published anonymously in this article. Ethical approval was obtained from our institutional review board before the study started (Date August 12, 2016/No. NL56144.018.16).

Each patient underwent CT scanning of both the affected and healthy contralateral forearms. Scans were used for 3D preoperative planning and for postoperative evaluation at approximately 6 weeks and 6 months in order to evaluate initial and final positioning and consolidation, respectively. The scans were carried out using a Brilliance 64-channel CT scanner (Philips Healthcare, Best, The Netherlands; isotropic voxel spacing 0.45 mm, 120 kV, 150 mAs, Pitch 0.6).

### Preoperative planning

**Fig. 1:**
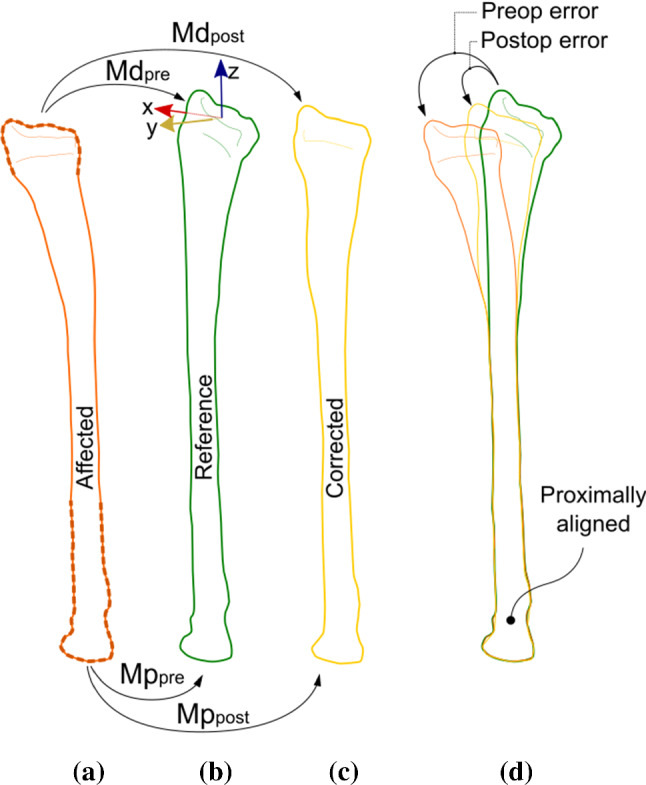
**a** Preoperative planning starts by segmenting the affected bone and clipping of a distal and a proximal segment. Registration of these segments to the image of the target bone (**b**) (see text for details) and to the postoperative image containing the corrected bone (**c**), provides a set of positioning matrices, which enable proximal alignment of the bones (**d**) and 3D quantifying of the preoperative and postoperative alignment error of the distal segment. The anatomical coordinate system (ACS) in (**b**) shows the radioulnar axis (*x*), dorsovolar axis (*y*) and proximodistal axis (*z*)

Preoperative planning began by carrying out a CT scan of both the affected and the contralateral bones for position planning. The contralateral bone in the mirrored image served as the reference bone, although a manual adaptation was sometimes needed to compensate for bilateral differences in length [8, 14]. Custom-made software [13] was used for position planning and evaluation in 3D. In short, the procedure works as follows: The affected radius was segmented from the preoperative CT scan. The reference radius was subsequently obtained by segmenting the contralateral bone from the mirrored image. Segmentation is based on a Laplacian level-set segmentation growth algorithm that was initialized by the result of threshold-connected region-growing of the cortical bone followed by hole filling. If needed, this procedure was alternated with manual painting. The Marching cubes algorithm [26] was used to extract a polygon mesh at the zero-level of the distance map that resulted from the level-set algorithm. This polygon mesh represented a virtual bone model (Fig. 1a). A distal and proximal bone segment was subsequently manually clipped from the affected bone model, thereby excluding the bone deformity. These segments were aligned by registration to the mirrored image of the contralateral healthy bone to find the target position (Fig. 1b). This provided a 4 × 4 homogeneous positioning matrix for the distal bone segment, **M**d_pre_, and the proximal bone segment, **M**p_pre_. These matrices are used to quantify the preoperative malalignment, as will be discussed below.

**Fig. 2:**
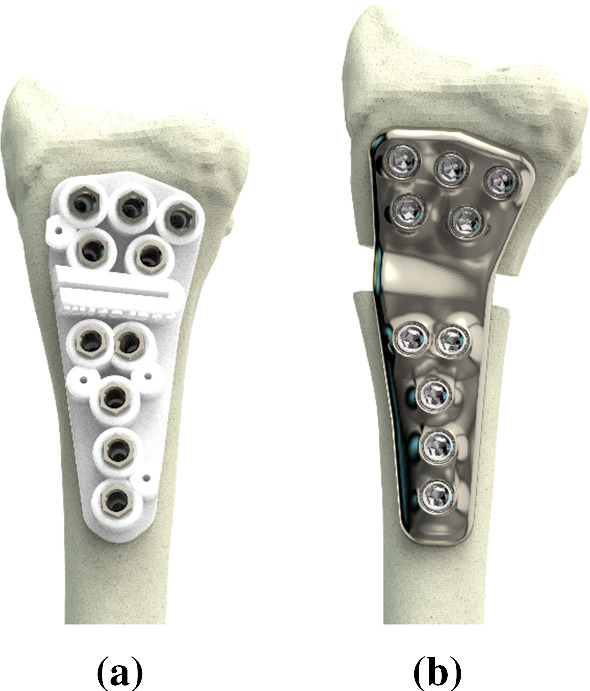
Example (patient #9) showing **a** drilling/cutting guide, and **b** a patient-specific plate and the planned position of the distal segment

During preoperative planning, we further chose locations for osteotomy and screw placement. A patient-specific polyamide drilling/cutting guide and a plate were subsequently designed (Fig. 2) [10], and the digital files were sent to Xilloc Medical for further processing and 3D titanium printing (Xilloc Medical, Sittard-Geleen, The Netherlands). All patient-specific instruments were sterilized at our institute.

### Surgical procedure

The drilling/cutting guide was fitted to the bone. Kirschner wires were used for temporary fixation of the guide, after which screw holes were drilled into the bone. The bone was cut using an oscillating surgical saw, first partially through the slit in the guide and then entirely after removal of the guide. After aligning the bone segments, the PSP is subsequently affixed, first to the distal bone segment using 2.4-mm locking screws (DuPuy Synthes, Raynham, MA) [27], and then to the proximal bone segment, if necessary using a Hintermann K-wire distractor.

Immobilization of the wrist was achieved using either a pressure bandage or a plaster cast, depending on patient preference. After surgery, the patients were instructed to keep the hand mobile by performing regular exercises but without exerting any additional external load, such as lifting. All patients started hand therapy on average 6 weeks after surgery, and received additional hand therapy if insufficient progress was observed.

### Clinical outcome measures

Hand function was assessed preoperatively and 6 months postoperatively by measuring range of motion parameters: flexion, extension, pronation, supination, radial deviation, ulnar deviation and grip strength. Grip strength was assessed using a Jamar™ dynamometer. Patients were asked to complete the Michigan Hand Outcomes Questionnaire (MHOQ) and the EuroQol EQ-5D-5L questionnaire (Dutch version) to rate their overall quality of life, expressed by an index in the range 0–1 (higher is better), and an overall Visual Analog Scale alternative (EQ-VAS) in the range 0–100 (higher is better).

### Radiographic outcome measures

Using **M**p_pre_, we transformed the aforementioned distal and proximal bone segments to the target image containing the mirrored contralateral bone (Fig. 1a, b). This aligns the bones proximally, while the distal segments show the preoperative positioning error (Fig. 1d). Registration of these bone segments to the corrected bone in the postoperative CT scan provides the postoperative registration matrices **M**d_post_, **M**p_post_ (Fig. 1a, c). These matrices enable quantifying the preoperative and postoperative positioning error (Fig. 1d) [13], while the planned position serves as the gold standard. Deviations from this plan are reported as positioning errors and are expressed in terms of three translations along, and three rotations about the axes of an anatomical coordinate system for the reference bone (ACS), as shown in Fig. 1b. This ACS is defined for the proximally aligned target bone and is based on the three gravitation axes of its polygon mesh points and defines the proximodistal axis, the ulnoradial axis, and the dorsovolar axis. The residual error was further quantified as the total translation error (TTE) defined as the root of the squared sum of the three translations described above, and as the total rotation error (TRE) defined as the root of the squared sum of the three rotations.

### Statistics

Given the small sample size, we used nonparametric methods for statistical testing. A Wilcoxon signed-rank test for paired samples was used to evaluate the null hypothesis of the patient-specific method to improve positioning and clinical results. To evaluate if clinical parameters correlated with malalignment parameters, a Spearman’s product moment correlation coefficient (rho) was determined as well as its level of significance after pooling preoperative and postoperative results. A significance level of *p* < 0.05 was used throughout this study. JASP version 0.12 (JASP Team, Amsterdam, The Netherlands) was used for statistical analysis.

## Results

**Fig. 3:**
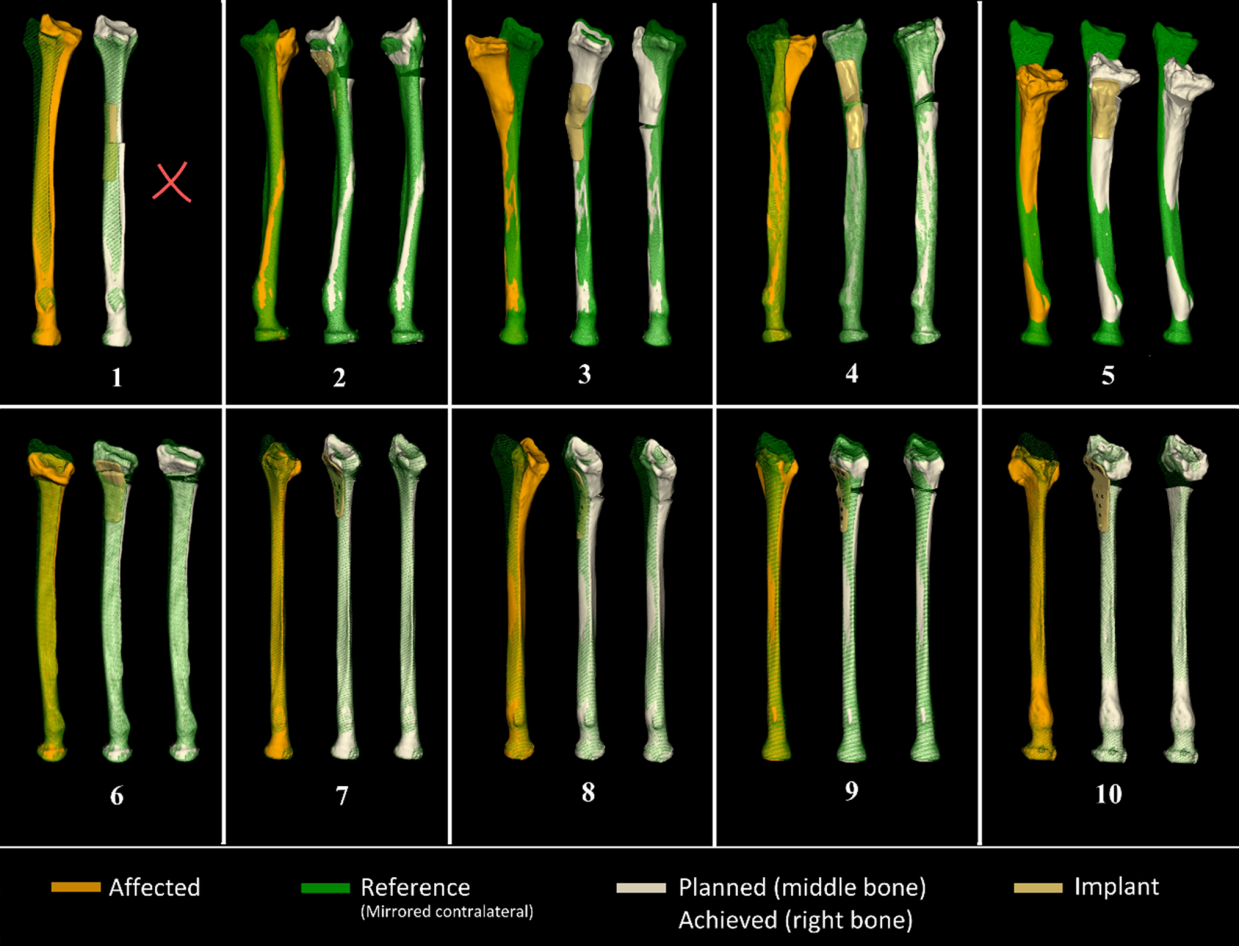
Ten patient cases included in this study showing, in triplets (from left to right): the deformed radius (orange), the planned alignment of the bone segments (white) showing the position and artist’s impression of a custom plate (gold), and the postoperatively achieved position of these bone segments (white). The proximally aligned green bone represents the mirrored contralateral bone as used in alignment planning

Patient characteristics are shown in Table 1. The average age of patients at surgery was 37 (SD 20), 6 (60%) were female, and average follow-up time for clinical and position evaluation was 6 months. The deformed bone (left: orange), planned alignment (middle: white with gold plate), and alignment achieved postoperatively (right: white without plate) in the PSP group are shown in triplets in Fig. 3, ordered by date of surgery. The superimposed radiuses (green) represent the mirrored contralateral bones and enable the visual assessment of the malalignment. Patient #1 did not return for a postoperative CT scan.

**Table 1:** Patient characteristics

Case #	Age (yrs)	Gender	Dominance (#)	Height (cm)	Weight (kg)	BMI	Smoker (*)	Affected side
1	44	M	L/R	192	80	21.7	C	L
2	73	F	R	171	69	23.6	P	R
3	20	M	R	189	105	29.4	C	L
4	41	F	L/R	165	84	30.9	N	R
5	18	M	R	187	78	22.3	N	L
6	45	F	R	170	62	21.5	N	L
7	36	F	R	163	54	20.3	N	L
8	16	F	R	176	98	31.6	N	L
9	16	M	R	185	61	17.8	N	L
10	64	F	R	171	62	21.2	N	L

(*) N, does not smoke; P, smoker in the past; C, currently a smoker; (#) L/R indicates ambidexterity

In three of the first four cases, the 2.4-mm Synthes locking screws broke at the plate boundary, either during surgery when applying a torsional load while testing mobility (case #1) or during daily activities after approximately six weeks (case #3 and #4). In these cases, conventional treatment followed in which a standard plate was used for bone fixation. Given this issue, the plates for the remaining six patient cases (#5–#10) were designed for, and fixed with, 3.5-mm Synthes locking screws instead of their 2.4-mm counterparts. No screw breakage complications occurred after implementing this change to the surgical approach.

In five other cases (#2, #5, #6, #7, #10), secondary surgery was performed at an average of 13 months after the initial surgery. Four of these cases (#2, #6, #7, #10) were for hardware removal of plate and screws, and one patient (#5, see Fig. 3) preferred secondary corrective surgery to facilitate lengthening of the radius with standard plating. During one hardware removal case (#7), the surgeon noticed damage to the flexor pollicis longus tendon and subsequently performed tendon repair. For case-specific details, see Table 2.

**Table 2:** Treatment details per case

Case #	Indication	Complexity (*)	Gap filling	Complications (till May 2020)
1	Congenital rotation deficit	+	Single cut No gap	Screws broke during surgery
2	Severe deformation with shortening and extra-osseos bone formation after Colles fracture	+ +	Donor bone and croutons	Hardware removal after 25 months
3	Malunion after antebrachii fracture with angulation and DRUJ instability	+ +	Donor bone and croutons	Screws broke after 3 months
4	Malunion radius shaft, excessive extra-osseous bone formation distally	+	Donor bone and croutons	Screws broke after 4 months
5	Extremely underdeveloped radius due to an epiphysiolysis in prepuberty and subsequent failed distraction osteotomy	+ + +	Donor bone and croutons	Secondary osteotomy after 15 months
6	Severe deformation with shortening and extra-osseos bone formation after Colles fracture	+ +	Donor bone and croutons	Hardware removal after 9 months
7	Distal radius malunion with bone deformation and shortening	+	No graft	Hardware removal after 6 months
8	Malunion radius shaft, with large deformation and DRUJ instability	+ +	Donor bone and croutons	−
9	Distal radius shaft fracture with supination deficit	+	Donor bone and croutons	−
10	Severe deformation with shortening and extra-osseos bone formation after Colles fracture	+ +	Donor bone	Hardware removal after 10 months

The cases in which screws broke are not included in the statistical evaluation (Table 3)

(*) As judged by the surgeon. + moderate, +  + severe, +  +  + extreme

**Fig. 4:**
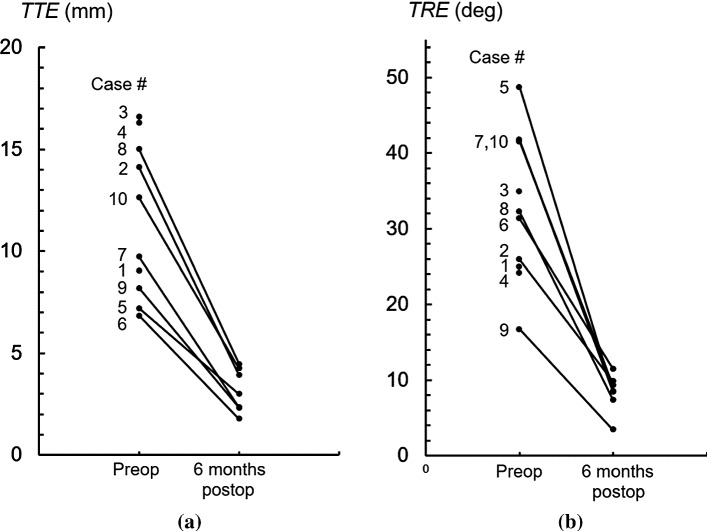
**a** Total translation error (TTE) and **b** total rotation error (TRE), showing the initial preoperative and residual 6-month postoperative malalignment. The connecting lines link error values on a case-by-case basis

Consolidation was confirmed by means of the 6-month postoperative CT scan. In patient #1, consolidation was confirmed using X-ray imaging. The preoperative and postoperative alignment errors were quantified by means of CT scan. To this end, the six-month postoperative CT scan was used (*N* = 7) or the 6-week postoperative CT scan in cases where the screws broke (*N* = 2, cases #3 and #4). Figure 4 shows lines connecting the pre- and postoperative alignment errors on a case-by-case basis. In subsequent statistical analyses, the screw breakout cases were excluded since these patients underwent secondary surgery without a PSP, leaving *N* = 7 PSP cases. Table 3 shows quantitative results (median and IQR) of a series of positioning and clinical parameters. In this table, the overall translation (TTE) and rotation parameters (TRE) are subdivided into their three directional counterparts. Significant repositioning improvements were mostly explained by translation in the proximodistal direction (*p* = 0.016) and rotations about the radioulnar axis (*p* = 0.016). Pain and overall outcome of the hand (MHOQ) were significantly improved after using the PSP (*p* = 0.018 and *p* = 0.031, respectively).

**Table 3:** Preoperative and postoperative parameter values (N = 7, unless indicated otherwise) represented by the median value and inter-quartile range (IQR)

Parameter	Preop	Postop	Significance *p*
*Positioning*			
Residual Translation Error, RTE (mm)	9.7 (7.7, 13.4)	3.0 (2.3, 4.1)	0.016 (*)
Residual radioulnar translation (mm)	2.2 (1.0, 4.1)	1.0 (0.6, 1.3)	0.109
Residual dorsovolar translation (mm)	4.4 (2.7, 9.1)	2.1 (1.4, 3.1)	0.109
Residual proximodistal translation (mm)	6.5 (6.2, 7.3)	1.6 (0.8, 2.1)	0.016 (*)
Residual Rotation Error, RRE (deg)	32.2 (28.6, 41.6)	8.5 (7.9, 9.5)	0.016 (*)
Residual radioulnar rotation angle (deg)	31.0 (21.8, 31.7)	4.1 (3.3, 7.7)	0.016 (*)
Residual dorsovolar rotation angle (deg)	4.4 (2.8, 6.6)	2.8 (1.2, 5.0)	0.469
Residual proximodistal rotation angle (deg)	8.8 (4.9, 18.8)	3.7 (2.4, 5.1)	0.078
*Clinical*			
Pain	3.0 (3.0, 4.0)	2.0 (2.0, 2.0)	0.018 (*)
Affected hand outcome (MHOQ)	44.7 (39.7, 58.4)	66.7 (59.2, 84.5)	0.031 (*)
Generic health status, index score (EQ-5D-5L)	0.73 (0.63, 0.75)	0.84 (0.82, 0.86)	0.142
Visual Analog Scale (EQ-VAS)	75.0 (62.5, 90.0)	85.0 (77.5, 92.5)	0.170
*Functional*			
Grip strength (Yamar) (*N* = 6) (kg)	17.5 (13.0, 21.2)	25.4 (21.7, 26.2)	0.063
Flexion–extension ROM (deg)	95.0 (72.5, 125.0)	100.0 (85.0, 132.5)	0.866
Pronation–supination ROM (deg)	95.0 (60.0, 110.0)	150.0 (100.0, 167.5)	0.058
Radioulnar deviation ROM (*N* = 6) (deg)	45.0 (25.0, 50.0)	50.0 (42.5, 50.0)	0.168

*p* < 0.05 indicates that the postoperative state is significantly different from the preoperative state

(*) Indicates statistical significance *p* < 0.05

To investigate the relationship between clinical and malalignment parameters, we pooled preoperative and postoperative results (*N* = 19, patient #1 did not return for the postoperative CT scan). A significant positive correlation was found between pain and malalignment of the distal segment (TTE: rho = 0.613, *p* = 0.007; TRE: rho = 0.604, *p* = 0.008; dorsovolar translation: rho = 0.660, *p* = 0.003; proximodistal translation: rho = 0.655, *p* = 0.003). Several parameters also showed a significant correlation with dorsovolar angulation of the distal segment (pain: rho = 0.616, *p* = 0.006; pronation–supination range, in percent of the healthy side: rho =  − 0.625, *p* = 0.004; MHOQ score: rho =  − 0.539, *p* = 0.017; Quality of life index EQ-5D-5L: rho =  − 0.512, *p* = 0.025). Finally, significant correlations were found between pronation and supination range, in percent of the healthy side and the TRE (rho =  − 0.486, *p* = 0.035), and between the MHOQ score and proximodistal translation (− 0.586, *p* = 0.008).

## Discussion

In this study, we evaluated the use of a patient-specific osteosynthesis plate for transferring a preoperative 3D plan to the patient. Our proposed approach has the advantage of combining both navigated positioning and bone segment fixation by using a PSP. This could ultimately simplify bone repositioning and render the procedure more accurate and precise in comparison with alternative methods or conventional corrective osteotomies, which are based on 2D position planning using radiographic parameters (radial inclination, volar tilt, ulnar variance). To be able to judge if positioning using a PSP performs better when compared with the conventional technique based on 2D imaging, we performed a groupwise Mann–Whitney *U* test with data from one of our previous studies [8] in which the residual positioning error of the conventional technique was evaluated. The comparison shows that positioning using a PSP performs significantly better (*p* < 0.001) than traditional treatment with a standard anatomical plate; at TTE = 6.8 mm and TRE = 17.5° for traditional treatment and TTE = 3.1 mm, TRE = 8.3° for the PSP approach. This observation is of the utmost importance, given the fact that the clinical outcome correlates with malalignment [8]. We also showed that our patients benefited from the treatment as their pain was reduced and overall hand function was improved.

Our study further demonstrated a correlation between pain and malalignment parameters (TTE, TRE, dorsovolar translation, proximodistal translation, and dorsovolar angulation). Many clinical parameters also correlate with dorsovolar angulation of the distal segment (pain, pronation–supination range, MHOQ score and quality of life index EQ-5D-5L). In a previous study, a correlation between clinical parameters and malalignment parameters was also established (pain vs. rotation about radioulnar axis; extension motion vs. rotations about radioulnar and proximodistal axes) [8]. Volar angulation was found to decrease the pronation–supination range of motion [28], which is in line with the correlation that we found between the dorsovolar angle and the pronation–supination range. Crisco et al. [29] found that dorsally angulated distal radius malunions showed altered DRUJ mechanics represented by a reduced joint space area, its repositioned centroid position, and lengthening of the radioulnar ligaments. These findings may explain the correlation with pain that we found, and may be related to the development of early degenerative joint disease. A positive ulnar variance often leads to wrist pain and is sometimes a reason for ulnar shortening after distal radius malunion [30]. This fact is also in agreement with the correlation that we found between pain and proximodistal translation. The correlations that we found are not surprising given the fact that the patients initially attend the clinic with symptoms of the wrist related to the deformity. On the other hand, the correlation proves that proper positioning is relevant and that techniques are needed that restore positioning as well as possible.

A number of different techniques have been proposed to transfer a preoperative 3D positioning plan for the distal radius to the patient. Athwal et al. proposed using tool-tracking equipment [9]. Their study setup was similar to ours in regard to evaluating positioning errors and clinical outcomes. However, in their study an optical tracking system was used to guide the surgical drill and saw intraoperatively, and a standard plate was subsequently used for fixation. Since their standard plate does not follow the exact bone contour, there could be a space between the plate and the bone surface, introducing the possibility of a positioning error. Their results seem promising, with residual errors of 1 mm and 2 degrees. However, these are mean values evaluated using 2D radiography. The error may be much higher in the individual patient as the variability of the method is not reported. Moreover, a 2D evaluation does not reveal a possible rotation error about the bone axis. Therefore, the true reliability of the method remains unknown. Another interesting technique was proposed by Murase et al. [18]. In their study, they inserted parallel pin pairs in the distal and proximal bone segments and performed the osteotomy using an initial guide, after which they navigated the bone segments by their pin pairs to the reduced position. This position can be maintained during surgery either by using a reduction guide [18], or a pre-adjusted external fixator device [13]. Unfortunately, 2D radiographic evaluation was also used in the study by Murase [18] and in a similar study by Michielsen et al. [31] to quantify radial inclination, volar tilt, and ulnar variance, instead of quantifying the positioning error in all six degrees of freedom. Therefore, the actual positioning error remains unknown. The same methodology was later used by Vlachopoulos et al. [32] who applied 3D evaluation and found an overall residual 3D rotation angle for opening and closing wedge osteotomy cases of 8.30 ± 5.35° and 3.47 ± 1.09°, respectively. The residual rotation error, i.e., about the proximodistal axis, was 3.73 ± 3.88° for opening wedge osteotomies, which implies a misinterpretation of the angles as observed in 2D posteroanterior and lateral radiographs. Translation errors in opening and closing wedge osteotomy cases were 2.00 ± 0.77 mm and 1.95 ± 1.93 mm. Three-dimensional evaluation of the method was also used by Roner et al. [33], who found an overall 3D rotation error of 5.8 ± 3.6° and a translation error of 1.6 ± 0.7 mm. These reported residual errors are comparable with those we found in our study (TTE = 3.1 mm, TRE = 8.3°).

Titanium PSPs have been 3D printed for a multitude of clinical applications, including reconstructive surgery of the skull [34], orbital floor [35], mandibula [36], maxilla [37], sternum [38], acetabulum [39], radius and ulna shaft [25], and distal radius [16, 24, 25]. Comparing the positioning results in applicable studies is often difficult since different evaluation parameters and dimensions are used to quantify the residual positioning error. Ciocca et al. describe mandibular reconstruction using a custom cutting guide and custom plate and report a residual frontal plane rotation of 1.28° around the unaffected condyle as evaluated using pre- and postoperative 3D models, which caused the corrected mandible to be 2.28 mm lower than planned [36]. Byrne et al. describe corrective osteotomies of both forearm bones at the distal level in one patient and at the middle-third level in four patients in which custom osteotomy guides and plates were used [25]. Radiographs (2D) were used for position evaluation, which hide rotation about the long axis of the bone. The residual positioning errors were 1.8° (range, 0.3 ± 5.2°) for the ulna and 1.4° (range, 0.2 ± 3.3°) for the radius. In a previous study, we presented a single patient case in which a custom cutting guide and plate were used for corrective osteotomy of the distal radius [16]. The defect was filled with a custom porous titanium implant. Residual translations in the radioulnar, dorsopalmar, and proximodistal direction were − 1.2, 0.4 and 0.4 mm. Residual errors for dorsopalmar flexion, radioulnar deviation, and supination–pronation rotations were − 0.9°, − 2.3° and 1.7°. Oka et al. [24] documented the results of a multicenter study of 16 upper extremity cases, including five humerus malunions, three diaphyseal malunions, and eight distal radius malunions and reported rotation errors of 0.73 ± 1.75°, 0.35 ± 0.88°, and − 0.70 ± 1.06° around, and translation errors of 0.04 ± 0.46 mm, − 0.10 ± 0.54 mm, and − 0.07 ± 0.57 mm along the *X*, *Y*, and *Z* axes of a local coordinate system. The residual positioning errors that we found postoperatively (TTE = 3.1 mm, TRE = 8.3°) were in the same order of magnitude as in the aforementioned studies but seem slightly larger compared to the results of Oka et al. [24]. The difference in the residual positioning errors may be explained by our shallow guides (Fig. 2a), which did not always fit to profound landmarks on the bone surface. This may have compromised fitting of the guide and hence bone positioning. Oka’s proposed solution for this issue was inserting a K-wire through the guide to penetrate the styloid in an attempt to confirm correct guide placement by means of intraoperative fluoroscopy. For future application, we recommend using guides that fit better to the bone surface, using a guide wire as in [24] or using extended guides [40].

Although the results of this case series seem promising, there are several limitations worth mentioning. The generalizability of our study may be limited due to a possible bias since a surgeon selected those cases that were eligible for treatment by PSP. We recommend carrying out a randomized controlled trial for further evaluation of the method and to quantify more parameters such as the ease of surgery and the overall costs including possible revision surgery and aftercare. This could confirm the finding of Bauer and coworkers [41] who reported that the use of patient-specific instruments helped to reduce the time of surgery, which we could not investigate in our study. We also did not report long-term follow-up results, which may affect postoperative complications, such as the number of revision surgeries. The relatively short follow-up time may also explain why we did not find a significant improvement in functional parameters (Table 3). This may further improve over time as shown in [24] where the final follow-up was one year. We did not perform a full biomechanical analysis and therefore did not take the optimal positioning of screws into account during the PSP design phase, which may explain the breaking of screws in three of the first four patient cases. Changing the screw diameter from 2.4 to 3.5 mm resolved this issue. In a parallel study [42], we investigated implant stress distributions and compared these in custom and standard distal radius plates. Under mechanical load, the highest stress levels were observed at the boundary of the plate and the screws, i.e., exactly at the location where the screws broke in our patients. Since custom plates are more rigid than standard anatomical plates [42], the stress level at the plate-screw boundary is higher, which may explain plastic deformation and breaking of screws at a lower load. Fatigue due to repetitive stress could very well be the causative mechanism that led to screw breakage, exactly at the location that we saw in these three patients. In cases #3 and #4, the screws broke after more than 3 months, which may be the result of prolonged stress in the screws as a result of slower bone healing, ultimately leading to fatigue. Another point of attention when using PSPs is the fact that it is not possible to adapt the plan if soft tissues resist stretching to the planned bone alignment, which was the case in patient #5 who required secondary corrective surgery.

## Conclusion

We have shown that preoperative pain, range of motion, MHOQ, and EQ-5D-5L scores correlate with malalignment of the distal bone segment. The PSP approach has been shown to improve bone alignment and reduce pain. It has further been shown to improve bone alignment in a way comparable to what can be achieved when using sophisticated 3D planning with a drilling/cutting guide, and the subsequent reduction guide. Overall, it performed better than conventional treatment solely based on planning using X-ray images and intraoperative fluoroscopic 2D evaluation.
